# Pre-septal and Orbital Cellulitis: A Retrospective Analysis of Manifestations and Outcomes of a Tertiary Center in Kuwait

**DOI:** 10.7759/cureus.65104

**Published:** 2024-07-22

**Authors:** Jafar Hayat, Lulwa Al-Musallam, Deemah Al-Shaya, Essa Tawfeeq, Mutlaq Al-Sihan, Raed Behbehani

**Affiliations:** 1 Department of Otolaryngology, Zain Hospital, Kuwait City, KWT; 2 Department of Ophthalmology, Al-Bahar Hospital, Kuwait City, KWT

**Keywords:** chandler classification, prevalence study, retrospective, orbital abscess, preseptal orbital cellulitis

## Abstract

Introduction

Orbital cellulitis is an infectious condition that presents a gap in the literature in regard to its manifestations in the Middle East. A retrospective investigation was undertaken at one tertiary care facility in Kuwait, aiming to augment regional insights into the etiological factors and subsequent outcomes associated with this condition.

Methods

A retrospective review collected data from 92 patients in a tertiary care center in Kuwait for patients admitted with pre-septal and orbital cellulitis between January 2013 and June 2023. Primary outcomes assessed the resolution of symptoms, categorized as "completely resolved" and "resolved with complications". Secondary outcome measures included patients’ ages, microbiology, laboratory, and radiological findings. Gender, predisposing conditions, surgical status, and orbital involvement were additionally extracted and analyzed descriptively.

Results

Data of n=92 (56 males and 36 females) were extracted. Ages ranged from 20 days to 18 years, the mean being seven years. Additionally, 52.2% (n=48) were diagnosed with orbital cellulitis, and 42.4% (n=39) were diagnosed with pre-septal cellulitis on admission. Cultures were available for n=60. Specifically, 52.1% (n=48) received CT scans; 6.5% (n=6) MRI scans; and 98.9% (n=91) received antibiotics (microbiology investigated: n=83). Ceftriaxone was used in 82.6% (n=76); 20.6% (n=19) received surgical interventions, and incision and drainage (I&D) were done on 15.2% (n=14). Visual acuity was assessed in 59.7% (n=55) patients. Five (5.43%) reported visual deterioration, and 87 (94.6%) of the outcomes were classified as “completely resolved”.

Conclusion

Upper respiratory tract infections were the most common documented predisposing risk factor in the development of pre-septal and orbital cellulitis. Adverse outcomes in vision were documented in 5.43% of patients. A paucity of regional data highlights the importance of conducting further studies locally.

## Introduction

Orbital cellulitis is characterized by infection of the orbit affecting tissues located posterior to the orbital septum beyond the orbital septum and extending into the orbital cavity. It carries both sight-threatening and potentially life-threatening morbidity [1,2]. It can manifest in all age cohorts; however, it predominantly presents in the pediatric population, and its successful treatment requires prompt recognition [3].

The most common organisms causing orbital cellulitis include Staphylococcus aureus and Streptococcus. Infection is most commonly associated with underlying sinusitis but is also caused by dental infection; pre-septal cellulitis progression, age, trauma, and foreign bodies [2].

Chandler’s classification categorizes orbital cellulitis based on severity and anatomical involvement. It is divided into five stages, each representing a varying level of severity. Chandler group 1 is classed as pre-septal cellulitis, while 2-5 are considered post-septal cellulitis, with group 1 being described as edema limited to the eyelid, 2 being inflammation including contents posterior to the septum, 3 being purulent collection between the bony orbit and periorbita, 4 being purulent collection within the orbit itself, and 5 being retrograde phlebitis [4].

Although the mainstay of treatment of orbital cellulitis is intravenous antibiotics, surgical drainage of abscesses may be necessary to relieve pressure and prevent further complications or lack of response to medical treatment [5,6].

There are few studies describing the clinical profile and outcome of orbital cellulitis in countries in the Arabian Gulf. Prior to this study, there exists a study of the microbiology profile of orbital cellulitis population in Saudi Arabia [7] in 2022, a chart review from 1991 to 2005 from a tertiary center in Riyadh [8], and a Saudi Arabian chart review published in 1989 [9].

We sought to describe the clinical manifestations and outcomes of periorbital cellulitis in one tertiary care center in Kuwait and to compare the findings with the clinical features and outcomes of pre-septal and orbital cellulitis outcomes reported in the literature.

## Materials and methods

A retrospective chart review of patients who were diagnosed with positive findings pertaining to the Chandler classification of orbital infections was conducted in Al-Bahar Hospital after obtaining ethical approval from the Kuwaiti institutional review board. The department was able to collect data from individuals admitted to the tertiary care center in Kuwait between the calendar years of 2013-2023, and the department was then able to extract and analyze patient characteristics, investigations, management, and outcomes of patients within these years.

Inclusion Criteria

We have included any patient who was admitted under a preliminary diagnosis of pre-septal cellulitis and orbital cellulitis (Chandler stages 1-5) between January 2013 and June 2023 in an Al-Bahar ophthalmology tertiary care center in Kuwait.

Exclusion Criteria

We excluded patients with insufficient medical records who were not admitted to this health center within the aforementioned timeframes.

Primary Outcome Measures

Our primary outcome measure evaluated the resolution of the conditions, which we defined resolution as the cessation of clinical signs and symptoms at discharge. We categorized outcomes as "completely resolved" and "resolved with complications”. Patients with reduced visual acuity and documented adverse outcomes were identified and labeled as “resolved with complications”.

Secondary Outcome Measures

Secondary outcome measures include evaluating patients’ ages, causative microorganisms, and radiological investigations (CT orbit/MRI scans).

Gender, hospitalization status, ocular findings, etiologic causes, risk factors, immune status, and orbital involvement were also collected. Relevant data were extracted and descriptively summarized to highlight trends in the region.

Admitted patients all underwent adequate ophthalmologic assessments for the sequelae of the disease.

Intervention

Any conservative, medical, or surgical intervention that patients underwent to manage pre-septal or orbital cellulitis.

Statistical Analysis

Descriptive statistics were employed to delineate demographic profiles, discern risk factors, and ascertain prevalence rates for various symptomatic, diagnostic, and management variables.

Risk of Bias Assessment

In assessing the risk of bias for this retrospective chart review, several potential sources of bias were considered. Selection bias was mitigated by including all eligible patient records from the specified time frame, ensuring a representative sample. To address information bias, data extraction was performed using a standardized protocol, with multiple reviewers cross-checking the data to enhance accuracy and consistency. Confounding variables, such as patient demographics and co-morbidities, were identified and extracted appropriately. To prevent reporting bias, all relevant outcomes and findings were transparently reported.

Funding and Conflicts of Interest

This research was conducted independently, without any financial support from external funding agencies, commercial entities, or non-profit organizations. Furthermore, the authors declare that they have no competing interests or conflicts of interest that could have influenced the outcomes or interpretations presented in this study.

## Results

We have included 92 patients admitted to Al-Bahar Hospital, with 48 being diagnosed with orbital cellulitis, 39 with pre-septal cellulitis, three with subperiosteal abscesses, two with orbital abscesses, and zero with cavernous sinus thrombosis on admission. The gender characteristics of the patients are represented in Table 1. The ages ranged from 20 days old to 18 years old. The mean age that was extracted was seven years old. The gender demographics of the set entailed 56 males and 36 females. 

**Table 1:** Genders of patients included in the study

Gender	Number of Patients	%
Male	56	60.9%
Female	36	39.1%

The predisposing variables to the disease are presented in Table 2. The most common predisposing condition was upper respiratory tract infection (URTI), present in 44.5% (n=41) patients. The second post-predisposing condition was having a fever of over 38c, present in 21.7% of patients (n=20).

**Table 2:** Predisposing conditions of patients on presentation

Predisposing Condition	Number of Patients	%
Upper Respiratory Tract Infection	41	44.56%
Fever (Unspecified)	20	21.73%
Sinusitis	12	13.04%
Trauma	12	13.04%
Bronchitis	1	1.09%
Otitis Media	3	3.26%
Viral Conjunctivitis	2	2.17%
Dacryocystitis	1	1.09%

Culture results were documented in 60 (65.2%) of patients. Findings were extracted and are available in Table 3 of the study. CT scans were obtained in 52.2% (n=48) and 6.52% (n=6) of the patients who received both CT and MRI scans. Additionally, 98.9% (n=91) of patients received IV antibiotic treatment on admission. Microbiological investigations including the culture of specimens were performed, and results were documented in 90.2% (n=83) patients. The most common antibiotic used was ceftriaxone, used in 82.6% of patients (n=76), with 41.3% (n=38) of the patients using it exclusively. Ceftriaxone combined with either clindamycin, metronidazole, vancomycin, or dexamethasone in varying combinations was also documented in 41.3% (n=38) of cases. Clarithromycin was also a documented treatment in four cases, amoxicillin/clavulanic acid in four cases, and clindamycin alone was used for three patients.

**Table 3:** Microbiology findings and frequency MRSA: Meticillin-resistant Staphylococcus aureus

Conjunctival Cultures	Number of Patients	%
No growth	38	41.3%
MRSA	2	2.17%
Staphylococcus epidermidis	2	2.17%
Coagulase-negative Staphylococcus	1	1.09%
Streptococcus pyogenes	1	1.09%
Haemophilus influenzae	2	2.17%
Staphylococcus haemolyticus	1	1.09%
Staphylococcus aureus	2	2.17%
Moraxella catarrhalis	1	1.09%
Blood Cultures	Number of Patients	%
No growth	2	2.17%
Staphylococcus epidermidis	1	1.09%
Acinetobacter lwoffii	1	1.09%
Nostril Cultures	Number of Patients	%
MRSA	1	1.09%
Coagulase-negative Staphylococcus	1	1.09%
Pus Swab Cultures	Number of Patients	%
No growth	1	1.09%
Pseudomonas aeruginosa	1	1.09%
Abscess Cultures	Number of Patients	%
Staphylococcus hominis	1	1.09%
Corneal Swab Cultures	Number of Patients	%
Haemophilus influenzae	1	1.09%

Additionally, 20.65% (n=19) of patients received surgical interventions (Table 4). The most common surgical interventions were incisions and drainages in 14 patients, followed by functional endoscopic sinus surgery in eight patients.

**Table 4:** Surgical intervention performed and distribution FESS: Functional endoscopic sinus surgery; I&D: Incision and drainage

Surgical Intervention	Number of Patients	%
Incision and drainage (alone)	10	10.9%
FESS (alone)	4	4.35%
Syringe and probing	1	1.09%
FESS and I&D	2	2.17%
FESS and I&D and orbitotomy	2	2.17%

Symptomatic resolution of orbital cellulitis occurred in 100% of patients (n= 92). Visual acuity was assessed in 59.7% (n= 55) of patients, and there was no change in visual acuity in 18 patients, while 32 patients reported improvements compared to vision on admission, and n=5 reported deterioration. Visual outcomes are available in Table 5. Clinical manifestations of individuals with acute visual deterioration are available in Table 6. Clinical manifestations of individuals with visual improvement during admission are available in Table 7.

**Table 5:** Vision outcomes compared to pre-intervention

Vision Outcomes	Number of Patients	%
No change	18	19.56%
Vision improved	32	34.78%
Vision deteriorated	5	5.43%
Not documented	37	40.22%

**Table 6:** Clinical manifestations of individuals with acute visual deterioration M: Male; F: Female; MRSA: Methicillin-resistant Staphylococcus aureus; IV: Intravenous; CT: Computerized tomography; MRI: Magnetic resonance imaging; VA: Visual acuity; RAPD: Relative afferent pupillary defect; EOM: Extraocular muscles; IOP: Intraocular pressure; OD: Right eye; RRR: Round, regular, and reactive to light; B/L: Bilaterally; R: Right; L: Left

Diagnosis	Age (Years)	Gender	Length of Admission	Vision at Presentation	Visual Acuity on Discharge	Ocular Symptoms at Presentation	Predisposing Conditions	Immune Status	Microbiology Results	Medical Management	Neuroradiological Findings	Managed Surgically
Left-Sided Orbital Cellulitis	8	M	5 Days	20/20 B/L	20/25 R, 20/40 L	A 1-day history of periorbital swelling, tenderness, and photophobia. A -1 restriction at upgaze and adduction was also identified.	Upper respiratory tract infection	Unremarkable	Conjunctival swab: MRSA.	IV Ceftriaxone IV Dalacin IV Dexamethasone	CT: Well-defined enhancing extraconal collection at the medial aspect measuring 9x21x19mm, extending to left anterior ethmoidal cells. Mild proptosis. Left preseptal soft tissue thickening/edema extending to lateral aspect of orbit.	No
Right-Sided Orbital Cellulitis	4	M	4 Days	20/30 B/L	20/40 R,20/40 L	A 3-day history of progressive periorbital swelling, watery discharge, pain, and fever. Right-sided conjunctival chemosis and erythema were also identified.	No	Unremarkable	Conjunctival Swab: Negative.	IV Ceftriaxone	CT orbit Impression: Subperiosteal Abscess.	No
Left-Sided Orbital Cellulitis	9	F	5 Days	20/20 B/L	20/20 R, 20/25 L	A 1-week history of left orbital swelling was on oral Amoxicillin + Clavulanic acid prior with slight improvements, the swelling, however, did not resolve. MRI done in private: Left orbital subperiosteal abscess with pansinusitis. Referred back to Al-Bahar; VA on admission: 20/20 bilaterally. Vision color 13/13 Bilaterally. On examination, the left eye showed ptosis and upper lid swelling. RAPD status:Negative. EOM: Limitation in superior rectus muscle; no proptosis. IOP: 12	No	Unremarkable	Left orbital abscess culture: No growth. Left conjunctival swab culture: No growth.	IV Ceftriaxone IV Clindamycin	CT Orbit Impression: Subperiosteal Abscess.	Underwent left subperiosteal abscess drainage under general anesthesia.
Right-Sided Orbital Cellulitis	13	M	8 Days	20/20 B/L	20/25 R, 20/20 L	A 1-day history of right-sided periorbital swelling and a 3-day history of a fever. Right-sided ptosis and proptosis +1; restriction in upgaze -2; upgaze and abduction -3; upgaze and adduction -2; adduction -1	Upper respiratory tract infection	Unremarkable	Pus culture: No growth. Blood Culture: Nogrowth.	IV Ceftriaxone IV Dalacin IV Dexamethasone	CT Orbit: Air fluid level with opaque bilateral maxillary, frontal, anterior ethmoidal sinuses; acute sinusitis. OD globe slightly proptotic with downward orientation and orbital collection seen adjacent to the orbital roof indenting the superior rectus; impression: subperiosteal abscess.	Underwent right orbital roof subperiosteal abscess drainage.
Right-Sided Orbital Cellulitis	12.5	M	8 Days	20/20 B/L	20/30 R, 20/20 L	A 4-day history of pain and erythema of the right eye; he was on Augmentin orally but had no improvement. Periorbital erythema was also identified on the right eye alongside pupil RRR.	History of the flu 3 weeks ago	Unremarkable	Conjunctival Swab: Negative.	IV Ceftriaxone IV Dalacin	CT Orbit: Right orbital cellulitis with a right subperiosteal collection and pansinusitis.	Bilateral Functional endoscopic sinus surgery and incision and drainage of the subperiosteal abscess alongside a bilateral concha bullosa decompression.

**Table 7:** Clinical manifestations of individuals with visual improvements during admission R: Right; L: Left; B/L: Bilateral; M: Male; F: Female; URTI: Upper respiratory tract infection; IV: Intravenous; CT: Computerized tomography; SPA: Subperiosteal abscess; MRI: Magnetic resonance imaging; RAPD: Relative afferent pupillary defect; PNS: Postnasal space; SPE: Superficial punctate erosions; EOM: Extraocular muscles; RRR: Round, regular, and reactive to light; URTI: Upper respiratory tract infection

Diagnosis	Age (Years)	Gender	Length of Admission	Vision at Presentation	Visual Acuity on Discharge	Ocular Symptoms at Presentation	Predisposing Illnesses	Immune Status	Microbiology	Medical Management	Neuroradiological Findings	Surgical Management
Left-sided orbital cellulitis	12	M	8 Days	20/20 R 20/40 L	20/20 B/L	A 1-day history of a -4 restriction in abduction, adduction, and elevation; -3 in depression	URTI and sinusitis	Unremarkable	No growth	IV Ceftriaxone IV Clindamycin IV Dexamethasone	CT Orbit: Left orbit: preseptal edema, fat stranding, extraconal tumefactive area (collection) with adjacent fat stranding seen superior to superior rectus muscle 3.1x2.9x2.6cm, compressing muscle causing globe displacement, with left-sided hyperdensity - could be debris. Another small linear hypodense collection of 2.4x1.4x0.2cm compressing and displacing the medial rectus. left-sided proptosis, swollen lacrimal gland. Evidence of bilateral maxillary, ethmoidal, frontal, sphenoidal sinusitis.	Underwent Incision and drainage of the abscess.
Left-sided preseptal cellulitis	10	F	2 Days	20/20 R, 20/30 L	20/20 R 20/25 L	A 1-week history of left-sided eye swelling and upper lid hyperemia, quiet eye with full ocular motility.	URTI	Unremarkable	No growth	IV Vancomycin IV Metronidazole IV Meropenem	Left preseptal cystic lesion. 3.7x2.5x2cm, with thickened irregular enhancing wall with internal septations, showing intra orbital extraconal extension, (features of orbital cellulitis with abscess) no intracranial extension. Mucosal thickening in maxillary + frontal sinus + partial opacification of ethmoid air cells + obliteration of maxillary ostia with mucosal thickening - features of sinusitis.	Underwent drainage of subperiosteal abscess.
Left-sided orbital cellulitis	12	M	9 Days	20/20 R, L 20/70	20/20 B/L	A 1-week history of left-sided eyelid swelling, gradually increasing in fullness. Subtotal ptosis, conjunctival echymosis, Left-sided -2 elevation, abduction, and adduction.	URTI	Unremarkable	No growth	IV Ceftriaxone IV Clindamycin IV Dexamethasone	CT Paranasal sinuses: Left-sided pansinusitis: totally opacified left ethmoid sinus and partially opacified left maxillary, frontal, sphenoid sinuses with mucosal enhancement. Medial rectus muscle bushed medially with small localized peripherally enhanced fluid collection 9.2 x 6.3 mm - lateral orbital wall related abscess.	Underwent a full-house functional endoscopic sinus surgery.
Right-sided orbital cellulitis	6	M	9 Days	20/30 R, 20/20 L	20/20 B/L	A 1 day history of peri-orbital swelling and pain on movement. Periorbital odema, conjunctival chemosis, and mild proptosis. A -2 restriction in elevation and abduction.	URTI	Unremarkable	No growth	Ceftriaxone Metronidazole Ephidrine nasal drops	Not done	None
Left-sided preseptal cellulitis+ subperiosteal abscess	13	F	4 Days	20/25 R 20/50 L	20/20 B/L	Complaints of pain and redness and swelling in the left eye alongside left eyelid swelling for 6 days and perioorbital swelling. Right eye swelling alongside upper eyelid swelling and ptosis.	None	Unremarkable	No growth	IV Ceftriaxone IV Clindamycin	CT orbit: Left Inflammatory thickening. A collection/abscess formation cannot be excluded. Massive sinonasal polyposis.MR orbit on 5/10: left preorbital preseptal inflammatory changes, associated with subperiosteal supra orbital abscess. Left apical and cavernous sinus changes.	SPA drainage, functional endoscopic sinus surgery.
Right-sided subperiosteal abscess	9	M	2Days	20/40 R, 20/50 L	20/20 B/L	Fever, proptosis, swelling, erythema of eyelid.	Chronic sinusitis	Unremarkable	No growth	IV Cefotaxime IV Vancomycin Switched later to IV Meropenem and Vancomycin	MRI brain: Intra-orbital abscess at the roof of the orbit, CT nasal paranasal sinuses: Mild regression of pansinusitis with intact bones.	Underwent a subperiosteal abscess drainage.
Left-sided orbital cellulitis	5.5	M	3 Days	20/25 R 20/70 L	20/25 R 20/30 L	A 4-day history of left eye swelling preceded by a 2-day history of fever, no history of trauma and worsening swelling. Upper left lid edema and hyperemia, ocular motility limitation in all directions mainly superotemporal, no RAPD.	Fever	Unremarkable	No growth	IV Clindamycin Augmentin syrup due to allergies	CT Paranasal sinuses: Sinusitis especially on the left side and with proptosis and orbital cellulitis with opacities over the left side.	None
Right-sided orbital cellulitis	7	M	5 Days	20/40 R 20/20 L	20/20 B/L	History of a headache followed by fever and proptosis. The patient was admitted by the pediatric department and a CT was done showing paranasal sinusitis with paranasal orbital edema. OD lid edema, proptosis, temporal limitation of movement, normal optic disc appearance, no papilledema.	Fever	Unremarkable	No growth	IV Ceftriaxone IV Metronidazole	CT orbit: Right paraseptal orbital cellulitis seen extending to the upper cheek with mild fat stranding. Evidence of pansinusitis. Right subperiosteal fluid collection and right proptosis.	None
Right-sided orbital cellulitis	8	M	7 Days	20/30 R 20/20 L	20/20 B/L	A 1-day history of fever and edema of the right upper eyelid.	URTI, fever	Unremarkable	Pus cultures: No growth	IV Vancomycin IV Ceftriaxone IV Metronidazole	CT Orbit: Right orbital proptotic changes. An extra conal soft tissue thickening extends along the superior and lateral walls of the right orbit, displaces the right eye globe and muscle cone inferiorly, a process associated with preseptal, prezygomatic thickening or edema. Minimal retrobulbar fat stranding at the right side. Near complete opacification of PNS. MRI brain: Status post-operaton revealing pansinusitis with right orbital paraseptal cellulitis. Right frontal epidural abscess and bifrontal focal meningitis.	Underwent an incision and drainage.
Right-sided orbital cellulitis	5	M	7 Days	20/25 R 20/20 L	20/20 B/L	Swelling of eyelids; tender on touch. ocular motility: -3 limitation in all directions. Proptosis; resistant to retropulsion	Fever	Unremarkable	Conjunctival swab: No growth	Iv Ceftriaxone IV Metronidazole	CT Paranasal sinuses: Subperiosteal soft tissue thickening (6mm in thickness) involving the medial aspect of the right orbital extraconal space, extending to the orbital apex and preseptal area alongside a localized collection (3mm in thickness) and secondary right globe proptotic changes.	Underwent functional endoscopic sinus surgery.
Bilateral orbital cellulitis	7	F	3 Days	20/80 R and 20/30 L	20/20 B/L	A 5-day history of progressive lid swelling and discharge. Right-sided proptosis, mechanical ptosis, lids tender and warm on touch, bilateral conjunctival chemosis, bilateral SPE, bilateral sluggish response of pupils. EOM: -4 in elevation, elevation and abduction, depression. Rest of EOM -3.	Pharyngitis, on and off history of tonsillitis. 5-day history of fever.	Unremarkable	Bilateral conjunctival swab: negative	IV Ceftriaxone	CT Orbit: Bilateral eye proptosis with the blurring of retro-orbital fat planes and ill-defined soft tissue thickening enshielding both sclera mainly posteriorly and extending along the distal part of the optic nerve. Mild diffuse preseptal thickening, mainly right-sided.	None
Right-sided orbital cellulitis	13	M	6 Days	20/20 R 20/30 L	20/20 B/L	The swelling started 2 weeks ago in the right eye with mild improvement with an Augmentin regimen of 1 gram x 10 days, then swelling increased again with pain in the right eye. Lid swelling and erythema, pupil RRR, full ocular motility, normal anterior segment.	Sinusitis	Unremarkable	No growth	IV Ceftriaxone IV Clindamycin	CT: Paranasal and maxillary sinusitis, right anterolateral abscess seen with CSF leak.	Functional endoscopic sinus surgery and right orbital abscess drainage and orbitotomy.
Right-sided orbital cellulitis	13	F	3 Days	20/50 R 20/40 L	20/40 R 20/20 L	A 1-week history of right-sided facial swelling with right lower lid edema. Right eye periorbital swelling with right eye ocular pain on movement. Proptosis, no chemosis, no RAPD.	URTI	Unremarkable	Conjunctival swab cultures: No growth, pus cultures: No growth	IV Ceftriaxone, Xylometazoline Nasal Spray Nasal steroid sprays, and normal saline nasal irrigation	CT Paranasal sinuses: Right maxillary sinus opacification, right sphenoid and ethmoidal mucosal thickening, right orbital floor abscess.	Patient underwent right lower lid transconjunctival abscess drainage under general anesthesia.
Right-sided orbital cellulitis	4	F	8 Days	20/40 R 20/20 L	20/30 R 20/20 L	A 3-day history of right eye swelling and pain. A history of gastroenteritis. Symptoms were progressive and not improving. Periorbital swelling, edema, redness and tenderness, clear cornea, no RAPD, pupil RRR.	URTI	Unremarkable	Right-sided conjunctival cultures: No growth	IV Ceftriaxone	CT Orbit: Bilateral maxillary and ethmoidal sinusitis, right ethmoidal mucocele with orbital involvement.	The patient underwent transnasal endoscopic drainage and ananterior orbitotomy.
Left-sided orbital cellulitis	15	M	11 Days	20/25 R 20/40 L	20/20 R 20/25 L	There was a gradual progressive upper lid of the left eye swelling for 2 weeks. Diplopia in upper gaze, upper lid erythema, conjunctival chemosis, RAPD: negative.	Sinusitis	Unremarkable	Pus swab cultures: pseudomonas aeruginosa	IV Ceftriaxone IV Metronidazole	CT Orbit: Left subperiosteal abscess and marked left sinusitis.	Functional endoscopic sinus surgery of the left side alongside a subperiosteal abscess drainage on the left side.
Right-sided orbital cellulitis	16	M	6 Days	20/30 R 20/20 L	20/20 B/L	Complaints of upper lid swelling with erythema for 4 days, preceded by flu 2 days before lid swelling. Right conjunctival chemosis, temporal, cornea clear.	Flu 2 days prior	Unremarkable	No growth	IV Ceftriaxone IV Metronidazole IV Clindamycin	CT Paranasal sinuses: Marked right preseptal edema and swelling and right sided cellulitis, a subperiosteal abscess, and pansinusitis.	None
Right-sided orbital cellulitis	15	M	8 Days	20/40 R 20/20 L	20/20 B/L	A 2-day history of pain, redness, and swelling of the eye. Limitation of outer movement. Redness, conjunctival chemosis, and mild proptosis were documented. Globe displacement by 4mm was documented.	None	Unremarkable	Right eye conjunctival swab: No growth	IV Clindamycin IV Cefotaxime IV Metronidazole IV	CT orbit: Signs of right orbital cellulitis and dacroadenitis were seen. Frontal, maxillary, and ethmoidal sinusitis.	None
Left-sided orbital cellulitis	12	M	6 Days	20/20 R 20/40 L	20/20 B/L	Complaints of left orbital swelling and pain for 1 week, symptoms worsened with febrile illness. Pain on left eye movement. Complete left-sided ptosis, chemosis, and pupil RRR.	Febrile illness	Unremarkable	Left eye conjunctival swab: No growth	IV Ceftriaxone IV Metronidazole	CT orbit: Left orbital cellulitis with left frontal ethmoidal and maxillary sinusitis with collection. Left orbital proptosis with preseptal swelling and edema. Right maxillary retention cyst.	None
Left-sided orbital cellulitis	14	M	5 Days	20/20 R 20/30 L	20/20 B/L	A 4-day history of lid swelling and the development of diplopia in all gazes. Lid erythema (warm to touch), proptosis, no RAPD, no chemosis, clear cornea.	URTI, sinusitis	Unremarkable	Conjunctival swab cultures: Coagulase-negative Staphylococcus	IV Ceftriaxone IV Clindamycin IV Dexamethasone	CT orbit: Left orbital cellulitis with post-septal extension and extraconal subperiosteal abscess and fluid collection, no intracranial extension, left pansinusitis.	Left functional endoscopic sinus surgery, left subperiosteal abscess drainage.
Left-sided preseptal cellulitis	6	F	5 Days	20/20 R 20/30 L	20/20 B/L	History of trauma with a metal object 2 days prior to presentation, since the trauma the patient developed redness, swelling and pain around the eye. Left-sided nasal deep wound with pus and discharge, left-sided periorbital swelling. No restrictions on eye movements.	Trauma	Unremarkable	Conjunctival eye swab: Streptococcus pyogenes	IV Ceftriaxone	Not done	None
Left-sided preseptal cellulitis	6	M	7 Days	R 20/30 20/50 L	20/20 B/L	A 3-day history of left lower lid edema, hyperemic upper lid, restriction of ocular motion in downgaze -1.	Fever and URTI	Unremarkable	Blood cultures: No growth	IV Ceftriaxone	Not done	None
Left-sided orbital cellulitis vs dacryoadenitis	9	M	5 Days	20/20 R 20/25 L	20/20 B/L	A 7-day history of left-sided bulging and pain of the eye, bulging and pain more localized laterally. Left-sided proptosis, upper lid tenderness, and swelling, EOM showed -2 elevation, -1 in abduction and adduction.	URTI	Unremarkable	Conjunctival cultures: No growth	IV Ceftriaxone and nasal decongestant	Not done	None
Left-sided orbital cellulitis	15	F	21 Days	20/70 R 20/200 L	20/20 B/L	A 2-week history of left eye orbital and sinus pain, associated with a 1-day history of blurring of vision, pain, redness, and upper lid swelling. Left-sided lid edema and mechanical ptosis, fundus showed disc swelling. Extraocular motility: Binocular diplopia in all gazes and limitation in all gazes -1 left-sided, limitation in abduction -2 and adduction -1 right-sided.	None	Unremarkable	Conjunctival swab: Negative	IV Ceftriaxone	CT Paranasal Sinuses: Soft tissue swelling behind the globe involving the optic nerve and extending superiorly in the orbit. No subperiosteal abscess was seen. Query orbital inflammatory syndrome vs papillitis. MRI done: findings suggestive of left optic neuritis.	None
Right-sided preseptal cellulitis	4	F	4 Days	20/60 R 20/60 L	20/40 B/L	A 1-day history of right upper lid swelling, increasing in size. Erythema, tenderness, and full ocular motility were documented.	None	Unremarkable	No conducted	Augmentin syrup, Cetirizine syrup.	Not done	None
Right-sided orbital cellulitis	6	M	1 Day	20/40 R 20/30 L	20/25 R 20/30 L	A 1-day history of right upper and lower lid swelling post-trauma 2 days prior. Right lid edema and tenderness and erythema. Quiet eye, ocular motility: Right-sided restriction in elevation, depression, abduction and adduction -1.	History of trauma 2 days prior to presentation, history of tonsillitis on treatment, history of URTI and fever	Unremarkable	Not conducted	IV Ceftriaxone IV Vancomycin	CT Paranasal Sinuses: Right-sided pan sinusitis, specifically ethmoiditis, with right orbital cellulitis displacing the medial rectus laterally including air bubbles.	Functional endoscopic sinus surgery.
Right-sided preseptal cellulitis	18	F	5 Days	20/25 R 20/20 L	20/20 B/L	A 3-day history of right-sided periorbital swelling. Lid edema, mechanical ptosis, conjunctival congestion, upper lid small abscess felt on palpation in the right upper temporal region that's draining pus; ocular motility full.	History of fever	Query hemophilia	Conjunctival swab: Staphylococcus aureus	IV Ceftriaxone	CT orbit: Right orbital cellulitis with post-septal extension and extraconal subperiosteal abscess and fluid collection, no intracranial extension.	The patient underwent incision and drainage for the abscess.

Admission dates sorted by month are available in Table 8. Eighty-seven (94.6%) of patients reported favorable outcomes and were classified as “completely resolved”, and n=5 (5.4%) reported visual loss upon discharge and were classified as “resolved with complications”.

**Table 8:** Admission frequency by month

Month of Admission	Number of Patients	%
January	10	10.9%
February	8	8.7%
March	10	10.9%
April	4	4.35%
May	9	9.78%
June	7	7.61%
July	4	4.35%
August	5	5.43%
September	3	3.26%
October	4	4.35%
November	11	11.96%
December	18	19.56%

## Discussion

Orbital cellulitis is an ocular emergency that requires urgent hospital admission due to complications that include vision loss, blindness, and even death [10]. This warrants urgent medical and surgical interventions [2] through the consultation of ophthalmologists and otolaryngologists. Both pre-septal and orbital cellulitis are major infections of the ocular adnexa and orbital tissues [11], with pre-septal cellulitis being defined as an infection anterior to the orbital septum and orbital cellulitis involving the tissues posterior to the orbital septum [12]. Diagnosis and management at times may prove to be a challenge for clinicians as manifestations such as induration, erythema, and edema are visually similar [13], with periorbital edema also limiting clinical examination and findings and hindering differentiability between the two conditions [4]. Differentiating cases that are clinically difficult to identify would warrant a CT paranasal sinus with contrast within 24 hours of presentation [14].

Whilst sex predilection is not expected to be observed in pre-septal and orbital cellulitis [15], 61% of cases observed in this study were male. November, December, January, and March were months with an increased number of observed presentations, secondary to increased URTI and sinus infections due to the weather [16]. The mean age in various studies ranges between four to eight years, which is what we observed in this study [17,18]. The frequency of pre-septal and orbital cellulitis in this study proved to be 68/32, differing from similar studies identifying a ratio of 87/13 in pediatric patients [17], a variance likely explained by regional referral practices.

Infective causes (upper respiratory tract infections, fevers (unspecified), and sinusitis) were the leading causes in a majority of cases in this study. Conjunctival cultures were the most common microbiology test ordered, with 50 ordered from reported data. No growth was found in 38/50 (76%), followed by a 2/50 (4%) positive culture for meticillin-resistant Staphylococcus aureus (MRSA) and a 2/50 (4%) positive culture for Haemophilus influenzae, a 2/50 (4%) positive culture for Staphylococcus epidermidis, and a 2/50 (4%) positive culture for S. aureus. This yields significantly different findings from the available literature, which demonstrated a 41/55 (75%) culture rate depicted in a study conducted by Liu et al. [19]. Of the microbiological microorganisms reported in this study, the most common were staphylococcus and streptococcus species, similar to what is reported in the literature [3].

Computerized tomography imaging is cited as the most utilized imaging modality in diagnosing sinusitis and evaluating orbital and subperiosteal abscesses [20,21]. Imaging was conducted on patients unresponsive to medical therapy or patients who are unable to be examined physically. The reported accuracy of CT imaging diagnosing abscesses ranges between 91% and 100% [20,22]. This study identified a total of 31 orbital and subperiosteal abscesses by utilizing CT scans, with the most common presenting complaint being either generalized swelling and patients not being able to open their eyes, serving as a reminder that initial presentations may not reveal occult abscesses revealed through radiological imaging.

Magnetic resonance imaging is the preferred imaging modality in identifying and excluding cavernous sinus thrombosis [23] and is more sensitive in evaluating intra-orbital complications of sinusitis [24]. This was utilized in six cases during the study, one of which identified optic neuritis, one with a sixth nerve palsy, and three with intra-orbital abscesses.

In this review, 91 patients underwent an empiric antibiotic regimen, with ceftriaxone being the most commonly prescribed monotherapy in 38 patients. Regimens were decided through symptomatic presentation and were prescribed prior to microbiology results. Similar studies discuss the validity of utilizing cephalosporins, aminoglycosides, and other combinations as appropriate antimicrobial regimens [2].

Surgical interventions were reserved for patients who had compromised visual function or were unresponsive to initial medical management, constituting 21% of patients in this study. This is a significantly higher figure than the 12.4% cited from a United States national study conducted in 2006 [25]. This may be attributed to differences in referral structures within local primary and secondary care centers. No reported patients had symptomatic recurrence and no intraoperative complications were reported in this study.

The strengths of this study include providing the first insights into documented characteristics of orbital cellulitis in Kuwait, providing both a perspective of characteristics and manifestations of the disease in the country and in the region. Other strengths include the high sample size of patients that were documented and the positive patient outcomes identified. Early retrospective studies in a region are significant as they provide a baseline on available data, management practices, and outcomes for future researchers to expand upon, providing valuable insights to those in the region.

The limitations of this study include the study's exclusively descriptive nature alongside its poor follow-up data due to the referral structures present in Kuwait. The study design could have also been optimized had it standardized microbiological investigations for every patient with a suspected positive admission diagnosis. Improvements to the data would also have been optimized with access to multiple primary and secondary center documentation systems, which is difficult to access secondary to the limited available data in certain primary and secondary care centers. This is a limitation for future researchers to address.

## Conclusions

Upper respiratory tract infections were the most common documented predisposing risk factor in the development of pre-septal and orbital cellulitis, followed by symptoms of fever and sinusitis. CT and MRI scans proved to be useful imaging modalities in investigating the condition. Approximately two-thirds of patients could be managed with IV antibiotic interventions with ceftriaxone being the most prescribed antibiotic overall.

A paucity of available data highlights the importance of conducting further studies locally and creating improved documentation systems in the region.
